# Insights into the European rabbit (*Oryctolagus cuniculus*) innate immune system: genetic diversity of the *toll-like receptor 3* (*TLR3*) in wild populations and domestic breeds

**DOI:** 10.1186/1471-2156-14-73

**Published:** 2013-08-21

**Authors:** Joana Abrantes, Helena Areal, Pedro J Esteves

**Affiliations:** 1 CIBIO/UP, Centro de Investigacao em Biodiversidade e Recursos Geneticos/Universidade do Porto, InBio, Laboratorio Associado, Campus Agrario de Vairao, Rua Padre Armando Quintas, number. 7, Vairao 4485-661, Portugal; 2 Departamento de Zoologia e Antropologia da Faculdade de Ciencias da Universidade do Porto, Porto, Portugal; 3 INSERM, U892, Universite de Nantes, Nantes 44007, France; 4 CESPU, Instituto de Investigação e Formação Avançada em Ciências e Tecnologias da Saúde, Gandra, Portugal

**Keywords:** Toll-like receptor 3 (*TLR3*), European rabbit (*Oryctolagus cuniculus*), RNA virus, Genetic diversity

## Abstract

**Background:**

Toll-like receptors (TLRs) belong to the innate immune system and are a major class of pattern recognition receptors representing the first line of the innate immune response. The TLR molecule is structurally composed by an ectodomain that contains leucine rich repeats (LRRs) that interact with pathogen associated molecular patterns (PAMPs), a transmembrane domain and a conserved cytoplasmic domain designated TIR (Toll-IL1 receptor) that is responsible for the intracellular signaling. TLR3 has been associated with the direct recognition of double-stranded viral RNA resulting from viral replication, while TLR7 and TLR8 target single-stranded viral RNA. In the European rabbit (*Oryctolagus cuniculus*), *TLR7* and *TLR8* were reported to be absent and pseudogenised, respectively, making TLR3 the only available TLR for the recognition of viral RNA. Thus, the levels of diversity of *TLR3* were evaluated in the European rabbit by analysing different genetic backgrounds and exposure to pathogen pressures.

**Results:**

We detected 41 single nucleotide polymorphisms (SNPs) in the coding sequence of *TLR3*. The highest diversity was observed in the wild populations of Iberian Peninsula, between 22–33 polymorphic positions. In the French population, 18 SNPs were observed and only 4 polymorphic positions were detected in the domestic breeds. 14 non-synonymous substitutions were observed, most of them in the LRR molecules. The remaining were scattered across the transmembrane and TIR domains.

**Conclusion:**

The study of TLR3 in European rabbit populations might be relevant to understand the interplay between RNA viruses and innate immunity. Wild rabbit populations presented more diversity than domestic breeds and other mammals previously studied. This might be linked to the absence of population bottlenecks during their evolution and to the almost inexistence of man-mediated selection. The observed variability might have also been potentiated by the contact of the wild populations with various pathogens. The study of these patterns of variability might reveal scenarios of host-pathogen interaction and identify *TLR3* polymorphisms’ that arose due to viral pathogens affecting wild populations.

## Background

The Toll like receptor (TLR) molecules are pattern recognition receptors (PRR) that mediate the recognition of pathogens by the innate immune system and can also lead to the activation of the adaptive immune response. To date, thirteen mammalian TLRs have been described that recognise pathogen associated molecular patterns (PAMPs), including bacterial lipoproteins, lipopolysaccharide (LPS) and viral nucleic acids [1]. TLR3 is triggered directly by double-stranded RNA (dsRNA) viruses or dsRNA resulting from their replication process [2]. The human TLR3 molecule is structurally characterised by the presence of an ectodomain that encompasses 23 leucine-rich repeats (LRRs) capped at each end by a LRR-NT and a LRR-CT molecule [3,4]. The LRRs main function is the interaction with PAMPs which in TLR3 occurs in the concave face of the TLR3-TLR3 homodimer [5]. In addition, human TLR3 has a transmembrane domain and a conserved cytoplasmic domain homologous to interleukin-1 receptor (IL1R) and IL-18 receptor (IL18R) designated TIR domain that is responsible for the intracellular signalling [5-7].

The lack of this receptor has been associated with higher susceptibility and mortality in infections by cytomegalovirus (CMV) and herpes simplex virus (HSV) in mice and humans [8,9]. On the other hand, some viruses, like West Nile Virus (WNV), benefit from the interaction with TLR3 that facilitates infection and dissemination in the organism [10]. In line with this disease association, several studies have pointed out a link between polymorphisms in TLR3 and resistance/susceptibility to diseases [11,12]. For instance, in humans, the *TLR3* single nucleotide polymorphism (SNPs) involved in the amino acid replacement N284I is responsible for the partial or total loss of activity of the receptor possibly by destabilising LRR architecture and the P554S SNP is associated with encephalitis caused by HSV [3].

In the present study a general approach was made to assess TLR3 diversity in wild and domestic European rabbits (*Oryctolagus cuniculus*) that have different genetic backgrounds and are exposed differently to pathogens. Viral TLRs (TLR3, 7, 8 and 9) act as the first line of defence against viral pathogens. However, in rabbit, *TLR7* and *TLR8* which are responsible for the recognition of ssRNA viruses are absent or pseudogenised, respectively [13], making TLR3 the only available TLR for the recognition of viral RNA.

**Figure 1:**
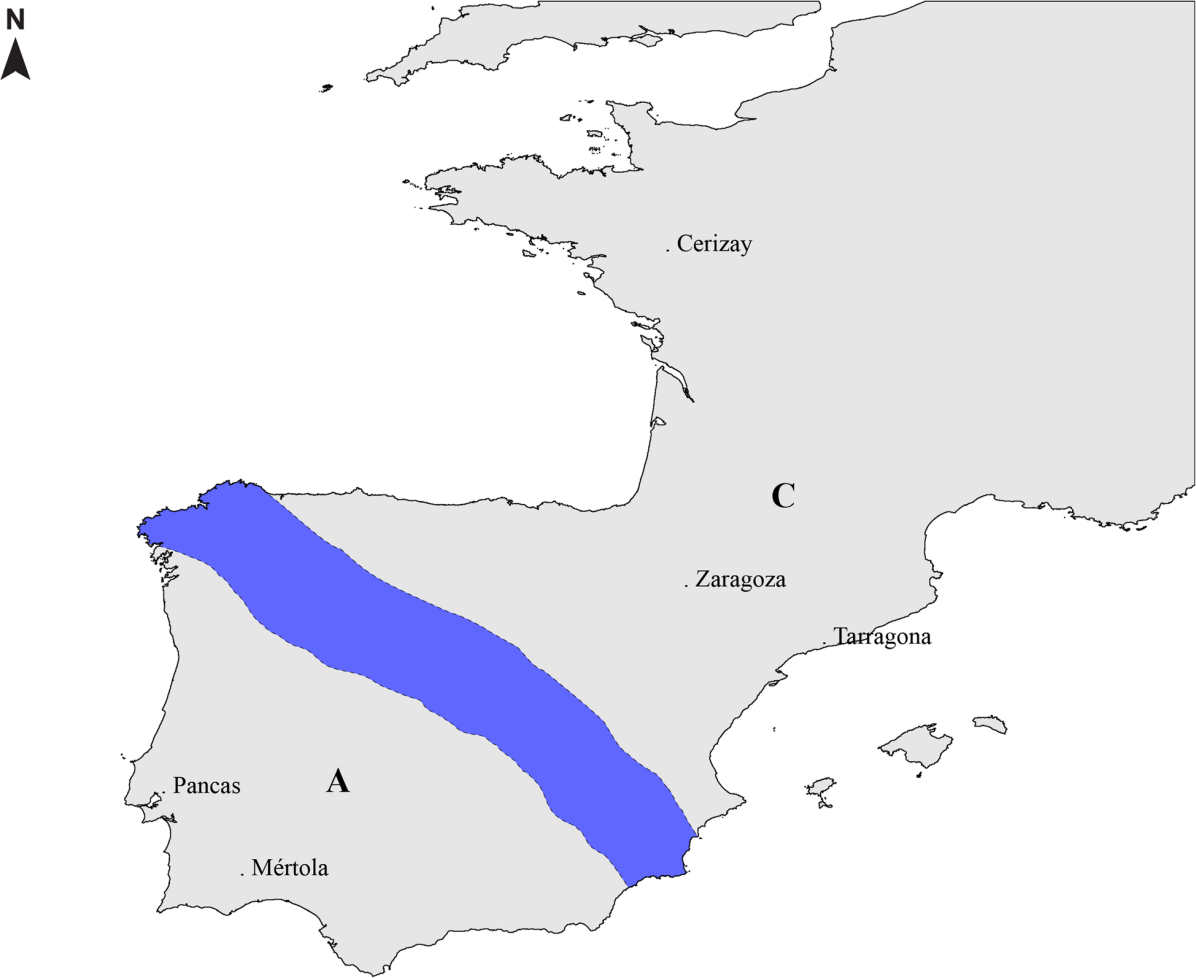
**Map with the geographical location of the populations used.** Blue shading represents the contact area between *O. c. algirus***(A)** and *O. c. cuniculus***(C)**.

The European rabbit originated in the Iberian Peninsula (IP) where two subspecies that diverged about 2 million years ago co-exist: *Oryctolagus cuniculus algirus* (OCA) in the Southwest part and *Oryctolagus cuniculus cuniculus* (OCC) in the Northeast part of IP [14] (Figure 1). More recently, the Northeast populations expanded to France and from these the species has been domesticated in the last 1,500 years with more than 200 breeds currently being recognised [15]. Thus, rabbit populations found outside the IP represent a subset of the original gene pool. Here, we have characterised genetically the TLR3 coding sequence in wild rabbits from the Iberian Peninsula and from France, and from domestic animals from 4 different breeds (Table 1) that represent different genetic backgrounds and which are exposed to different pathogen pressures aiming at finding polymorphisms that might interfere with the TLR3 function.

**Table 1:** List of the samples used including information regarding their subspecies/breed, country, sampling locality, number of individuals and reference to previous studies

**Subspecies (breed)**	**Country**	**Locality**	**Number of individuals**	**References**
*O. c. algirus*	Portugal	Pancas	10	[14,16,17]
		Mértola	10	[14]
*O. c. cuniculus*	Spain	Zaragoza	10	[14,16,17]
		Tarragona	10	[14,16,17]
	France	Cerisay	20	[18,19]
French Lop	n.a.	n.a.	5	[20]
English Spot	n.a.	n.a.	5	[20]
Argent Champagne	n.a.	n.a.	5	[20]
New Zealand	n.a.	n.a.	5	[20]

n.a. not applicable.

## Results and discussion

**Figure 2:**
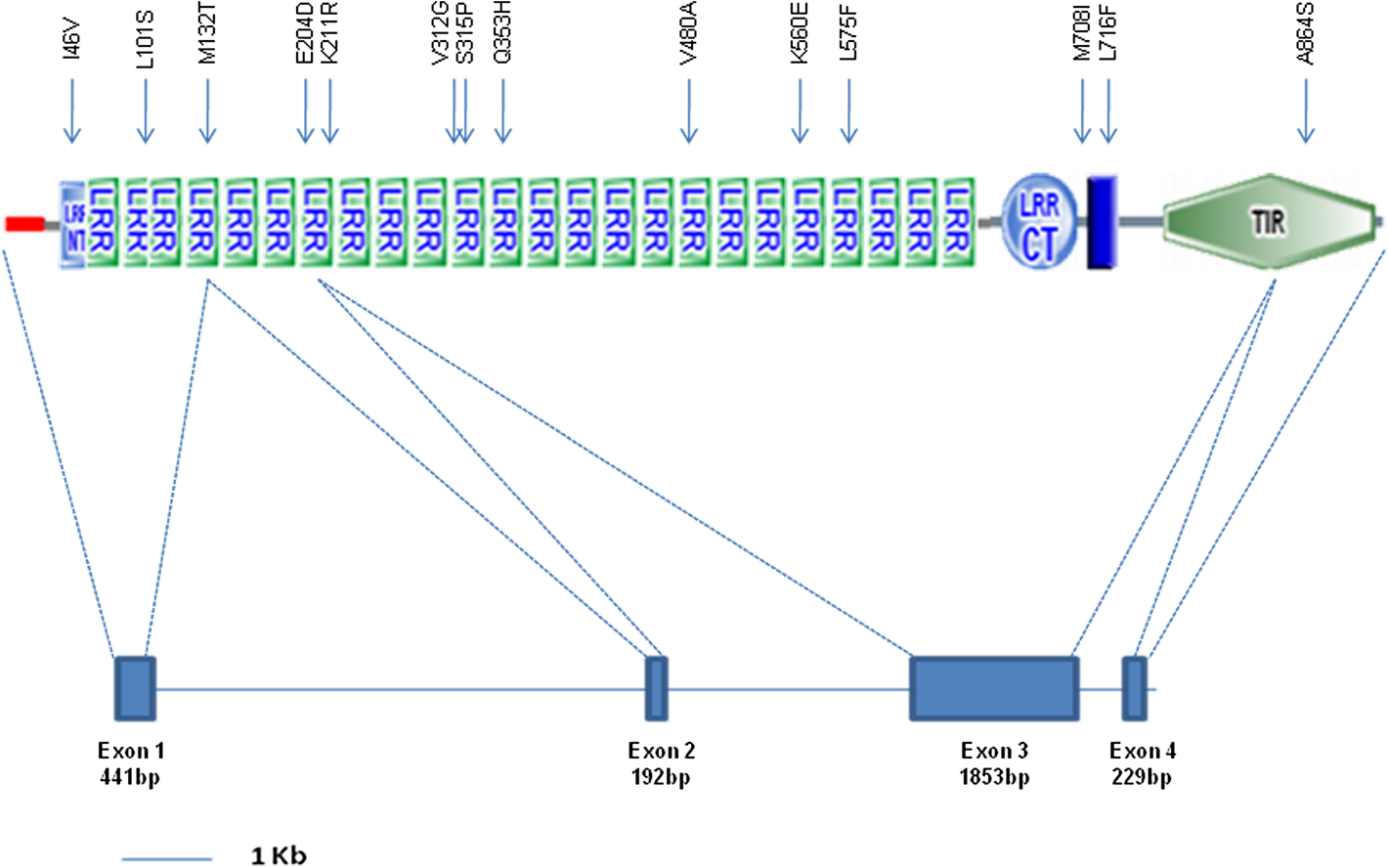
**Schematic diagram of the European rabbit TLR3 molecule domains.** Domains were adapted from SMART (http://smart.embl-heidelberg.de/) [21]. Non-synonymous substitutions are indicated on the top. The *TLR3* gene structure is presented at the bottom.

According to Ensembl (http://www.ensembl.org; ENSOCUG00000017763), *TLR3* is assigned to chromosome 2 of the European rabbit genome, with a total length of 11762 bp. The gene is organised into 4 or 5 exons with the coding sequence (CDS) comprising 2718 bp and 2655 bp, respectively. These transcripts translate into 905 and 884 amino acids, respectively, with the shorter transcript having a 21 amino acid deletion at exon 3 corresponding to amino acids 783–803. The analysis of the TLR3 structure revealed the presence of an ectodomain with a LRR-NT, twenty four LRRs and a LRR-CT, a transmembrane and TIR domains (Figure 2). This structure is in agreement to that of other TLRs.

Several studies addressing the *TLR3* genetic variation showed low levels of diversity in many mammals. Indeed, in nine breeds of bovines, seven SNPs were found of which three were non-synonymous [11]. In addition, in 84 male pigs from eleven breeds nine synonymous SNPs and six non-synonymous were detected [22] while in five horse breeds four SNPs were described of which two are non-synonymous [13]. Here we detected 41 SNPs in the coding sequence of *TLR3* of which 14 are non-synonymous (Additional file 1: Table S1). This discrepancy in values could be due to the inclusion of wild animals in the present study which were not considered in the species previously described, and which in turn might have contributed for the higher levels of diversity found here. In particular, the results showed higher diversity in the OCA populations where 33 polymorphic positions were observed, whereas only 22 were observed in OCC from IP, 18 in OCC from France and only 4 in the domestic breeds (Table 2). These results are further confirmed by the population genetic parameters summarised in Table 2. Even though no diagnostic positions were observed, some were exclusive to each population (Table 2). In addition, a gradient of genetic diversity loss in the same direction as the domestication process (from the Iberian populations to the French populations; Table 2) seems to occur as expected due to the bottleneck effect associated with this process [16]. However, the Tajima D’s test did not reject the null hypothesis that the populations evolve under neutrality (Table 2).

**Table 2:** **Levels of nucleotide sequence polymorphism in the populations/domestic studied for *TLR3***

	**N**	**S**	**π**	**D**
**All**	**160**	**41**	**0.0027**	**−0.0015**
*O. c. algirus*				
Portugal	40	33	0.0031	0.3310
Pancas	20	23	0.0023	−0.1429
Mértola	20	30	0.0035	0.4839
*O. c. cuniculus*				
Spain	40	22	0.0024	0.8565
Zaragoza	20	18	0.0022	0.7376
Tarragona	20	19	0.0023	0.6632
France	40	18	0.0025	1.9930
Domestic	40	4	0.0004	0.2620

N, number of sequences; S, number of segregating sites; π, nucleotide diversity [23]; D, Tajima’s D statistic test [24].

Seventy-five haplotypes were recovered of which the majority (64/75 ≈ 85%) are rare occurring only once in heterozigoty and only two (haplotypes 39 and 40) are shared between different populations (see Additional file 2: Table S2). Indeed, haplotype 39 is common in OCC from IP and France and all domestic breeds, which is expected as these are the closest populations. Haplotype 40 is more restricted and shared only by Iberian OCC from Tarragona and France and the English Spot breed. It is also interesting to note that OCA populations do not share haplotypes with the other subspecies neither among the two populations which reveal a high degree of genetic variability across individuals. With respect to the domestic individuals, all specimen from French Lop, Argent Champagne and New Zealand are characterised by presenting a single haplotype while in the English Spot breed 3 different haplotypes are present. The estimation of haplotype diversity showed a pattern of loss of genetic diversity from the Iberian to the French populations (Table 3). As expected, the lowest values were observed for the domestic breeds. The higher variability in the wild populations may be due to the absence of man-mediated selection and a greater exposure to pathogens. Additionally, the low genetic diversity observed for the French populations when comparing with the IP populations might be explained because they are a subsample of the Iberian OCC which has recently colonised France [20,25,26].

**Table 3:** **Estimates of haplotype diversity, haplotype diversity variance and haplotype standard deviation in the populations/domestic studied for *TLR3***

	**H**	**Hd**	**Hd variance**	**Hd std deviation**
**All**	**75**	**0.883**	**0.00051**	**0.023**
*O. c. algirus*				
Portugal	38	0.997	0.00004	0.006
Pancas	18	0.989	0.00037	0.019
Mértola	20	1.000	0.00025	0.016
*O. c. cuniculus*				
Spain	28	0.969	0.00025	0.016
Zaragoza	16	0.974	0.00063	0.025
Tarragona	13	0.911	0.00289	0.054
France	9	0.763	0.00227	0.048
Domestic	4	0.310	0.0077	0.088

H, number of haplotypes; Hd, haplotype diversity; Hd variance, variance of haplotype diversity; Hd std deviation, standard deviation of haplotype diversity.

Of the total number of polymorphisms, ≈ 34% (14/41) are non-synonymous substitutions that could be a source of protein variability that might interfere in the ability of TLR3 to recognise ligands and lead to resistance/susceptibility to viral diseases (Table 4). In particular, the variation observed at residues 480, 560 and 864 is unique to the European rabbit as these amino acids are generally conserved among mammals (data not shown). Although no data associate the observed substitutions with disease, it is likely that populations that are in contact with different pathogenic agents co-evolve with them and display more genetic diversity in order to confer resistance. Examples of two viral diseases responsible for high mortalities in domestic and wild European rabbits are RHDV and myxomatosis [27,28]. This variation could however be just the result of stochastic events that occurred during evolution of the protein.

**Table 4:** Amino acid variation found at TLR3 in each population/domestic

		**Position**													
**Subspecies**	**Population**	46	101	132	204	211	312	315	353	408	560	575	708	716	864
**ENSOCUT00000017763**		**I**	**L**	**M**	**E**	**K**	**V**	**S**	**Q**	**S**	**K**	**L**	**M**	**L**	**S**
*O. c. algirus*	Pancas	I	L/S	M	E	K/R	V	S	Q	V/A	K	F	M	L/F	A/S
	Mertola	I	L/S	M	E/D	K/R	V	S	Q/H	V	K	F	M/I	L/F	A/S
*O. c. cuniculus*	Tarragona	I/V	L/S	M/T	E	K	V	S/P	Q	V	K	F/L	M	L/F	A/S
	Zaragoza	I	L/S	M/T	E/D	K	V	S	Q	V	K/E	F/L	M	L/F	A
	Cerisay	I	L/S	M	E	K	V/G	S	Q	V	K	F/L	M	L/F	A
	Domestic	I	L	M	E	K	V	S	Q	V	K	F*	M	L	A

*only in the English Spot breed: F/L.

Regarding the location of the amino acid changes, most (11) are in the LRR molecules and the remaining (3) are distributed across the transmembrane and TIR domains (Figure 2). The higher variation in the LRRs is expected as these molecules are responsible for the direct interaction with PAMPs and should evolve to maintain recognition and trigger the innate immune response. The interaction between TLR3 and dsRNA occurs in two binding sites localised in the region comprising LRR-NT and LRR3 and LRR19-20 [3]. Of the non-synonymous substitutions observed in the LRRs, three are located within these interaction points. Nevertheless, none of these alterations (p.I46V in the LRR-NT, p.L101S in LRR2 and p.K560E in LRR20; Table 4) is conserved among vertebrates (data not shown) or essential for recognition [29,30]. LRR20, along with LRR21 and LRR22, are known for their importance in binding and signalling [31], but the p.L575F alteration observed in the LRR20 is conservative.

Other regions in the ectodomain have also been described as being important for ligand binding such as those between R65 to K163 and K330 to K493 that were described by Choe, Kelker et al. (2005) as a patch of positively charged residues [31]. In this region, of the four amino acid changes observed in this study, only one (p.Q353H; Figure 2 and Table 4) results in changes in the polarity. The importance of the ectodomain in the PAMPs recognition as well as the role of the possible coevolution with the pathogens they recognise in the evolution of this domain has been a recurrent matter particularly shown in the high level of positive selection in this domain across mammal TLRs [32]. This supports that the changes observed in the ectodomain of TLR3 might be the result of adaptation of TLR3 to maintain recognition of the evolving viral PAMPs. Nonetheless, none of the non-synonymous substitutions here detected is located within the sites detected previously as being under positive selection in mammals [32].

In the transmembrane domain, two amino acid changes were found, but both are conservative in terms of polarity and charge. In contrast, the alteration in the TIR domain, A864S (Figure 2 and Table 4), results in change of a hydrophobic residue for one hydrophilic corresponding to a possible site of N-glycosylation that may have consequences in the functionality of this domain. The TLR3 molecule is highly glycosylated in its ectodomain where there are 15 predicted N-glycosylation sites that affect ligand binding or control the access to the interaction surfaces [3]. Ligand binding in TLR3 only occurs at the glycan-free surface [31]. Despite this, the functional consequences of a possible N-glycosylation site in the TIR domain are unknown.

The biological consequences of each of the described alterations in the TLR3 molecule of the European rabbit also remains unknown, but further studies should be conducted to assess their impact since in this species only this TLR is available for detection of RNA viruses.

## Conclusions

The differences in the *TLR3* genetic variability detected between the wild rabbit populations and the domestic breeds reflects the domestication process of this species but might also result from the different exposure to pathogens. Indeed, while wild populations are usually in contact with a higher number of pathogens, domestic breeds are usually bred in highly controlled facilities. The higher number of non-synonymous substitutions found in the ectodomain, which corresponds to the binding surface to the PAMPs, further suggests a role of the host-pathogen interaction in promoting variability. In addition, the limited degree of variability found in the domestic breeds might compromise survival in face of a viral infection putting at risk the studied breeds.

## Methods

The program LRRfinder was used to determine the European rabbit TLR3 domain structure [33] (http://www.lrrfinder.com/).

In this study we analysed 20 individuals of the subspecies *Oryctolagus cuniculus cuniculus* from Spain (Tarragona (OCCIP_T) and Zaragoza (OCCIP_Z)) and 20 of the subspecies *Oryctolagus cuniculus algirus* from Portugal (Pancas (OCA_P) and Mertola (OCA_M)). These natural populations are located within the species’ original range. Twenty individuals from a French population (Cerisay (OCCF)) and 20 domestic animals (5 individuals per breed (DOM)) belonging to the French Lop, English Spot, Argent Champagne and New Zealand breeds were also studied (Table 1). DNA was extracted with the phenol-chloroform method [34]. Amplification was carried out with Taq DNA Polymerase (Taq PCR Master Mix Kit, Qiagen) with several sets of primers (see Additional file 3: Table S3). The thermal profile consisted of a denaturation of 95°C for 15 min and then, 30 cycles of 95°C for 30s; optimal annealing temperature for 30s; 72°C for 45 s and a final amplification at 60°C for 20 min. PCR amplicons of the expected size were sequenced with BigDye® Terminator v3.1 Cycle Sequencing Kit (Applied Biosystems, Foster City) in the following temperatures: 94°C for 3 min, 23 cycles at 96°C for 10s, 55°C for 5 s and 60°C for 4 min. The electropherogram analysis was performed in 3130xl Genetic Analyzer (Applied Biosystems, Foster City).

Sequences were edited, aligned and analysed with CLUSTAL W [35] as implemented in the BioEdit software [36]. Haplotype reconstruction was obtained for each individual using PHASE (Version 2.1) [37] as implemented in DnaSP (Version 5.10.01) [38]. Only haplotypes with P > 0.90 were considered as inferred reliably. Population genetic parameters (S, π), Tajima’s D, number of haplotypes, haplotype diversity, variance of haplotype diversity and standard deviation of haplotype diversity were estimated with DnaSP. The sequences obtained were also aligned and compared to the reference sequence available in Ensembl (ENSOCUT00000017763).

### Availability of supporting data

Data obtained in this study was submitted to GenBank under the following accession numbers: KC963181 to KC963340.

### Ethics for field studies

All sampling collection was conducted in accordance with local legislation and with the permissions and licences of the National institutions that supervise the hunting activities. The Convention on Biological Diversity and the Convention on the Trade in Endangered Species of Wild Fauna were respected.

## Abbreviations

TLR: Toll-like receptor; LRR: Leucine-rich repeats; PAMP: Pathogen associated molecular pattern; TIR: Toll-IL1 receptor; SNP: Single nucleotide polymorphism; PRR: Pattern recognition receptor; LPS: Lipopolysaccharide; dsRNA: Double-stranded RNA; IL1R: Interleukin 1-receptor; IL18R: Interleukin 18-receptor; CMV: Cytomegalovirus; HSV: Herpes simplex virus; WNV: West Nile virus; IP: Iberian Peninsula; OCA: *Oryctolagus cuniculus algirus*; OCA: *Oryctolagus cuniculus cuniculus*; CDS: Coding sequence.

## Competing interests

The authors declare that they have no competing interests.

## Authors’ contributions

JA and PJE conceived of the study, participated in its design and coordination and helped to interpret data and draft the manuscript. HA carried out the experimental work and analysed the data. All authors read and approved the final manuscript.

## Supplementary Material

Additional file 1: Table S1 List of the SNPs found for each population/breed and their location in the *TLR3* gene (positions are according to the CDS of ENSOCUT00000017763 from Ensembl). Click here for file

Additional file 2: Table S2 List of the *TLR3* haplotypes found with indication of their frequency and occurrence in the populations/breeds analysed. Click here for file

Additional file 3: Table S3 Amplification conditions of *TLR3*. Characterisation of the conditions used for *TLR3* amplification. Positions are according to the rabbit *TLR3* sequence (ENSOCUG00000017763). Click here for file
