# Supplementary material for: Insights into the European rabbit (Oryctolagus cuniculus) innate immune system: genetic diversity of the toll-like receptor 3 (TLR3) in wild populations and domestic breeds
Source: BMC Genet. 2013 Aug 21;14:73. doi: 10.1186/1471-2156-14-73 (PMC3844586; doi:10.1186/1471-2156-14-73)
Supplement: Additional file 1: Table S1 — List of the SNPs found for each population/breed and their location in the TLR3 gene (positions are according to the CDS of ENSOCUT00000017763 from Ensembl). [file 1471-2156-14-73-S1.doc]

**Table S1** **List of the SNPs found for each population/breed and their location in the** ***TLR3*** **gene (positions are according to the CDS of ENSOCUT00000017763 from Ensembl)**

| Subspecies | **Exon** | **1** | | | | | | | | **2** | | | | **3** | | | | | | | | | | | | | | | | | | | | | | | | | | **4** | | |
| --- | --- | --- | --- | --- | --- | --- | --- | --- | --- | --- | --- | --- | --- | --- | --- | --- | --- | --- | --- | --- | --- | --- | --- | --- | --- | --- | --- | --- | --- | --- | --- | --- | --- | --- | --- | --- | --- | --- | --- | --- | --- | --- |
|  | Position (bp) | 90 | ***136** | 243 | 246 | ***302** | 303 | ***395** | 408 | 585 | ***612** | 618 | ***632** | 810 | 822 | ***935** | 943 | ***988** | ***1059** | 1074 | 1143 | 1144 | 1281 | 1299 | ***1439** | 1606 | 1626 | 1656 | 1665 | ***1678** | ***1725** | 1765 | 1911 | 1953 | ***2124** | ***2148** | 2208 | 2229 | 2409 | 2505 | 2523 | ***2590** |
|  | **ENSOCUT00000017763** | **G** | **A** | **G** | **C** | **T** | **G** | **T** | **C** | **C** | **G** | **A** | **A** | **T** | **C** | **T** | **T** | **T** | **G** | **G** | **C** | **T** | **G** | **C** | **T** | **T** | **T** | **C** | **T** | **A** | **G** | **C** | **T** | **A** | **G** | **G** | **T** | **C** | **C** | **T** | **C** | **G** |
| *xO. c. algirus (IP)* | Pan2 |  |  |  |  |  |  |  |  | T |  |  |  | Y |  |  |  |  |  |  |  |  |  |  |  | C | C |  |  |  | **T** |  | C |  |  | **T** | C | T |  |  |  |  |
|  | Pan3 |  |  |  |  | **C** |  |  |  | T |  | M |  | C |  |  |  |  |  |  | Y | Y |  | S |  | C | Y | Y |  |  | **T** |  | C | R | **R** | **K** | Y | Y |  |  | Y | **K** |
|  | Pan5 |  |  |  |  |  |  |  |  |  |  |  |  | C |  |  |  |  |  | A | T | C |  |  |  | C | C |  |  |  | **T** |  | C |  |  |  |  |  |  |  |  |  |
|  | Pan6 |  |  |  |  |  |  |  |  |  |  |  |  |  |  |  |  |  |  |  |  |  |  |  |  | C | C |  |  |  | **T** |  | C |  |  | **T** | C | T |  |  |  |  |
|  | Pan7 |  |  |  |  | **C** |  |  |  | T |  | M | **R** | C |  |  |  |  |  |  | Y | Y |  | S |  | Y | Y |  |  |  | **T** |  | C |  |  | **K** | Y | T |  |  |  |  |
|  | Pan8 |  |  |  |  | **Y** |  |  |  |  |  | M |  | C |  |  |  |  |  |  |  | C |  |  |  | C | C |  | Y |  | **T** |  | C |  |  |  | Y | T |  |  |  |  |
|  | Pan10 |  |  |  |  |  |  |  |  | T |  | M | **R** | C |  |  |  |  |  |  |  | Y |  |  |  | C | C |  |  |  | **T** |  | C |  |  | **T** | C | T |  |  |  |  |
|  | Pan11 |  |  |  |  | **C** |  |  |  |  |  |  |  | C |  |  |  |  |  |  |  | Y |  |  | **Y** | C | C |  |  |  | **T** |  | C |  |  |  |  |  |  |  |  |  |
|  | Pan14 |  |  |  |  | **C** | A |  | A |  |  |  |  | Y |  |  |  |  |  |  |  |  |  |  |  | C | C |  |  |  | **T** |  | C |  |  | **T** | C | T |  |  |  |  |
|  | Pan20 |  |  |  |  | **C** | A |  |  | T |  |  | **R** | C |  |  |  |  |  |  |  | Y |  | S |  | C | C |  |  |  | **T** |  | C |  |  | **K** | Y | T |  | Y |  |  |
|  | Mer25 |  |  | R |  | **C** | A |  | M | T |  | M |  | C |  |  |  |  |  |  |  |  |  |  |  | C | Y | Y |  |  | **Y** |  | C |  |  | **K** |  |  | M |  |  | **K** |
|  | Mer26 |  |  | A |  | **C** | A |  | A | T |  | M |  | C |  |  |  |  |  | R | Y | Y |  |  |  | C | C |  |  |  | **T** |  | C |  |  | **K** | Y | Y |  |  |  |  |
|  | Mer27 |  |  |  |  |  |  |  |  | T |  |  |  | C |  |  |  |  |  |  | Y | C |  | G |  | Y | Y | Y |  |  | **T** |  | C |  |  |  |  | Y |  | Y |  |  |
|  | Mer29 |  |  |  |  | **C** | A |  | A |  |  |  |  | C | Y |  |  |  | **S** |  |  | Y |  |  |  | Y | Y | Y |  |  | **T** |  | C |  |  |  |  | T |  | C |  |  |
|  | Mer30 | S |  |  |  |  |  |  |  | Y |  |  |  |  |  |  |  |  | **S** |  |  |  |  |  |  | Y | Y | Y |  |  | **T** |  | C |  | **R** |  |  |  |  |  | Y |  |
|  | Mer31 |  |  |  |  | **C** | A |  |  | T |  | M |  | Y |  |  |  |  |  | R | Y | C |  | S |  | Y | Y | Y |  |  | **T** |  | C |  |  |  |  |  |  |  |  |  |
|  | Mer32 | C |  |  |  |  |  |  |  | T | **C** |  |  | C | Y |  |  |  |  |  | Y | C | R | S |  | C | C |  |  |  | **T** |  | C |  |  |  |  | T |  | Y |  |  |
|  | Mer34 |  |  |  |  | **C** | A |  |  |  |  | M | **R** |  |  |  |  |  |  |  |  | C |  | S |  | Y | Y | Y |  |  | **T** |  | C |  |  |  |  |  | M |  |  |  |
|  | Mer35 |  |  | A |  | **C** | A |  | A | T |  | M | **R** | C |  |  |  |  |  |  | Y | Y |  | S |  | Y | Y | Y |  |  | **T** |  | C |  |  | **K** | Y | Y |  |  |  |  |
|  | Mer36 |  |  |  | Y | **C** | A |  |  | Y |  |  |  | C |  |  |  |  | **S** |  | Y | Y |  | S |  |  |  | T |  |  | **T** |  | C |  |  |  |  |  |  |  | Y |  |
| *O. C. cuniculus (IP)* | Tar6,9 |  |  |  |  |  |  |  |  | Y |  |  |  |  |  |  |  |  |  |  | Y | Y |  |  |  |  |  |  |  |  | **S** |  |  |  |  |  |  |  |  |  |  |  |
|  | Tar10 | C | **G** |  |  |  |  |  |  | T |  |  |  | Y |  |  |  |  |  |  |  |  |  |  |  |  |  |  |  |  | **C** |  |  |  |  |  |  |  |  |  |  |  |
|  | Tar102 |  |  |  |  |  |  |  |  | T |  |  |  | Y |  |  |  |  |  |  | T | C |  |  |  | Y | Y |  |  |  | **C** |  | Y |  |  | **K** | Y | Y |  |  |  |  |
|  | Tar103 |  |  |  |  |  |  |  |  | T |  |  |  | C |  |  |  |  |  |  | T | C |  |  |  | C | C |  |  |  | **Y** |  | C |  |  | **T** | C | T |  |  |  |  |
|  | Tar105 |  |  |  |  |  |  |  |  | Y |  |  |  |  |  |  |  |  |  |  | T | C |  |  |  |  |  |  |  |  | **C** |  |  |  |  |  |  |  |  |  |  |  |
|  | Tar106 |  |  |  |  | **Y** |  |  |  |  |  |  |  |  |  |  |  |  |  |  |  |  |  |  |  | Y | Y | Y |  |  | **T** |  | C |  |  | **K** |  | Y |  |  | T | **K** |
|  | Tar107 |  |  |  |  | **Y** |  |  |  | T |  |  |  |  |  |  |  |  |  |  | Y | Y |  |  |  |  |  |  |  |  | **C** |  |  |  |  |  |  |  |  |  |  |  |
|  | Tar110 |  |  |  |  |  |  | **C** |  | Y |  |  |  | Y |  |  |  |  |  |  | Y | Y |  |  |  | C | Y |  |  |  |  |  | Y |  |  | **K** | Y | Y |  |  |  |  |
|  | Tar111 |  |  |  |  |  |  |  |  | Y |  |  |  |  |  |  | **Y** |  |  |  | Y | Y |  |  |  |  |  |  |  |  | **S** |  |  |  |  |  |  |  |  |  |  |  |
|  | Zrg2 |  |  |  |  |  |  |  |  |  |  |  |  |  |  |  |  |  |  |  |  |  |  |  |  |  |  | Y |  |  | **T** |  | Y |  |  |  |  |  |  |  | Y |  |
|  | Zrg4 |  |  |  |  |  |  |  |  | Y |  |  |  | Y |  |  |  |  |  |  | Y | Y |  |  |  |  | Y |  |  |  | **T** |  |  |  |  |  |  |  |  |  |  |  |
|  | Zrg5 |  |  |  |  | **Y** | R |  |  | T | **C** |  |  | C |  |  |  |  |  |  | Y | Y |  |  |  | C | Y |  |  |  | **T** |  | Y |  |  | **K** |  | Y |  |  |  |  |
|  | Zrg6 |  |  |  |  |  |  | **Y** |  |  |  |  |  |  |  |  |  |  |  |  |  |  |  |  |  |  |  |  |  |  |  |  |  |  |  |  |  |  |  |  |  |  |
|  | Zrg7 |  |  |  |  | **Y** | R |  |  | Y | **S** |  |  | Y |  |  |  |  |  |  | Y | Y |  |  |  | Y | Y | Y |  | **R** | **T** |  | C |  |  | **K** |  | Y |  |  | Y |  |
|  | Zrg8 |  |  |  |  |  |  | **Y** |  |  |  |  |  |  |  |  |  |  |  |  |  |  |  |  |  |  |  | Y |  |  | **T** |  | Y |  |  |  |  |  |  |  | T |  |
|  | Zrg10 |  |  |  |  |  |  |  |  |  |  |  |  | Y |  |  |  |  |  |  | Y | Y |  |  |  |  | Y |  |  |  | **T** |  | Y |  |  | **K** | Y | Y |  |  |  |  |
|  | Zrg11 |  |  |  |  |  |  |  |  |  |  |  |  |  |  |  |  |  |  |  |  |  |  |  |  | C |  |  |  |  | **S** |  |  |  |  |  |  |  |  |  |  |  |
|  | Zrg13 |  |  |  |  | **Y** | R |  |  | Y | **S** |  |  | Y |  |  |  |  |  |  | Y | Y |  |  |  | C | Y |  |  |  | **T** |  | Y |  |  | **K** |  | Y |  |  |  |  |
|  | Zrg14 |  |  |  |  |  |  |  |  |  |  |  |  |  |  |  |  |  |  |  |  |  |  |  |  | C |  | Y |  |  | **T** |  | Y |  |  |  |  |  |  |  | Y |  |
| *O. c. cuniculus (France)* | CY63B, 67 |  |  |  |  |  |  |  |  | T |  |  |  | Y |  | **K** |  |  |  |  | T | C |  |  |  | Y | Y |  |  |  | **Y** |  | Y |  |  | **K** | Y | Y |  |  |  |  |
|  | CY64, 140, 199, 202, 212 |  |  |  |  |  |  |  |  |  |  |  |  |  |  |  |  |  |  |  |  |  |  |  |  |  |  |  |  |  |  |  |  |  |  |  |  |  |  |  |  |  |
|  | CY65 |  |  |  |  |  |  |  |  | T |  |  |  | C |  | **G** |  |  |  |  | T | C |  |  |  | C | C |  |  |  | **T** |  | C |  |  | **T** | C | T |  |  |  |  |
|  | CY66, 75, 201, 126B, 167 |  |  |  |  |  |  |  |  | Y |  |  |  | Y |  | **K** |  |  |  |  | Y | Y |  |  |  | Y | Y |  |  |  | **K** |  | Y |  |  | **K** | Y | Y |  |  |  |  |
|  | CY72, 83 |  |  |  |  |  |  |  |  |  |  |  |  | C |  |  |  | C |  |  |  |  |  |  |  |  |  | T |  |  |  | T |  |  |  |  |  |  |  |  |  |  |
|  | CY18 | S |  |  |  | **Y** | R |  |  | T |  |  |  | Y |  | **K** |  |  |  |  | T | C |  |  |  | Y | Y |  |  |  | **Y** |  | Y |  |  | **K** |  | Y |  |  |  |  |
|  | CY137 | C |  |  |  |  |  |  |  | T |  |  |  | C |  | **G** |  |  |  |  | T | C |  |  |  | C | C |  |  |  | **T** |  | C |  |  | **T** | C | T |  |  |  |  |
|  | CY154 |  |  |  |  |  |  |  |  | Y |  |  |  | C |  | **K** |  | Y |  |  | Y | Y |  |  |  | Y | Y | Y |  |  | **K** |  | Y |  |  | **K** | Y | Y |  |  |  |  |
|  | CY156 |  |  |  |  |  |  |  |  | Y |  |  |  | Y |  | **K** |  | Y |  |  | Y | Y |  |  |  | Y | Y | Y |  |  | **K** |  | Y |  |  | **K** | Y | Y |  |  |  |  |
|  | CY206 |  |  |  |  |  |  |  |  | Y |  |  |  |  |  |  |  |  |  |  | Y | Y |  |  |  |  |  |  |  |  | **S** |  |  |  |  |  |  |  |  |  |  |  |
| *O. c. cuniculus (Domestic)* | FrL1, 2, 3, 4, 5; ArgC89, 13, 14, 15, 16; NZ17, 18, 19, 20, 21 |  |  |  |  |  |  |  |  |  |  |  |  |  |  |  |  |  |  |  |  |  |  |  |  |  |  |  |  |  |  |  |  |  |  |  |  |  |  |  |  |  |
|  | Eng7, 9, 11 |  |  |  |  |  |  |  |  | Y |  |  |  |  |  |  |  |  |  |  | Y | Y |  |  |  |  |  |  |  |  | **S** |  |  |  |  |  |  |  |  |  |  |  |
|  | Eng61 |  |  |  |  |  |  |  |  | T |  |  |  |  |  |  |  |  |  |  | T | C |  |  |  |  |  |  |  |  | **C** |  |  |  |  |  |  |  |  |  |  |  |
|  | Eng10 |  |  |  |  |  |  |  |  | Y |  |  |  |  |  |  |  |  |  |  |  | C |  |  |  |  |  |  |  |  | **S** |  |  |  |  |  |  |  |  |  |  |  |

*non-synonymous substitutions
