# Supplementary material for: Insights into the European rabbit (Oryctolagus cuniculus) innate immune system: genetic diversity of the toll-like receptor 3 (TLR3) in wild populations and domestic breeds
Source: BMC Genet. 2013 Aug 21;14:73. doi: 10.1186/1471-2156-14-73 (PMC3844586; doi:10.1186/1471-2156-14-73)
Supplement: Additional file 2: Table S2 — List of the TLR3 haplotypes found with indication of their frequency and occurrence in the populations/breeds analysed. [file 1471-2156-14-73-S2.docx]

**Table S2.** List of the *TLR3* haplotypes found with indication of their frequency and occurrence in the populations/breeds analysed.

| **Haplotytpe** | **Nucleotide Positions** | | | | | | | | | | | | | | | | | | | | | | | | | | | | | | | | | | | | | | | | | **Frequency** | **Population^*^** | |
| --- | --- | --- | --- | --- | --- | --- | --- | --- | --- | --- | --- | --- | --- | --- | --- | --- | --- | --- | --- | --- | --- | --- | --- | --- | --- | --- | --- | --- | --- | --- | --- | --- | --- | --- | --- | --- | --- | --- | --- | --- | --- | --- | --- | --- |
|  |  |  |  |  |  |  |  |  |  |  |  |  |  |  |  |  |  |  |  |  |  |  |  |  |  |  |  |  |  |  |  |  |  |  |  |  |  |  |  |  |  |  |  |  |
|  | 90 | 136 | 243 | 246 | 302 | 303 | 395 | 408 | 585 | 612 | 618 | 632 | 810 | 822 | 935 | 943 | 988 | 1059 | 1074 | 1143 | 1144 | 1281 | 1299 | 1439 | 1606 | 1626 | 1656 | 1665 | 1678 | 1725 | 1765 | 1911 | 1953 | 2124 | 2148 | 2208 | 2229 | 2409 | 2505 | 2523 | 2590 |  | |  |
| 1 | G | A | G | C | T | G | T | C | T | G | A | A | T | C | T | T | T | G | G | C | T | G | C | T | C | C | C | T | A | T | C | C | A | G | T | C | T | C | T | C | G | 1 | | OCA |
| 2 | . | . | . | . | . | . | . | . | . | . | . | . | C | . | . | . | . | . | . | . | . | . | . | . | . | . | . | . | . | . | . | . | . | . | . | . | . | . | . | . | . | 1 | |  |
| 3 | . | . | . | . | C | . | . | . | . | . | . | . | C | . | . | . | . | . | . | T | C | . | G | . | . | . | . | . | . | . | . | . | . | . | . | . | . | . | . | T | T | 1 | |  |
| 4 | . | . | . | . | C | . | . | . | . | . | C | . | C | . | . | . | . | . | . | . | . | . | . | . | . | T | T | . | . | . | . | . | G | . | G | T | C | . | . | . | . | 1 | |  |
| 5 | . | . | . | . | . | . | . | . | C | . | . | . | C | . | . | . | . | . | A | T | C | . | . | . | . | . | . | . | . | . | . | . | . | . | G | T | C | . | . | . | . | 2 | |  |
| 6 | . | . | . | . | . | . | . | . | C | . | . | . | . | . | . | . | . | . | . | . | . | . | . | . | . | . | . | . | . | . | . | . | . | . | . | . | . | . | . | . | . | 2 | |  |
| 7 | . | . | . | . | C | . | . | . | . | . | . | . | C | . | . | . | . | . | . | T | C | . | G | . | T | T | . | . | . | . | . | . | . | . | G | T | . | . | . | . | . | 1 | |  |
| 8 | . | . | . | . | C | . | . | . | . | . | C | G | C | . | . | . | . | . | . | . | . | . | . | . | . | . | . | . | . | . | . | . | . | . | . | . | . | . | . | . | . | 1 | |  |
| 9 | . | . | . | . | . | . | . | . | C | . | C | . | C | . | . | . | . | . | . | . | C | . | . | . | . | . | . | C | . | . | . | . | . | . | G | . | . | . | . | . | . | 1 | |  |
| 10 | . | . | . | . | C | . | . | . | C | . | . | . | C | . | . | . | . | . | . | . | C | . | . | . | . | . | . | . | . | . | . | . | . | . | G | T | . | . | . | . | . | 1 | |  |
| 11 | . | . | . | . | . | . | . | . | . | . | . | . | C | . | . | . | . | . | . | . | C | . | . | . | . | . | . | . | . | . | . | . | . | . | . | . | . | . | . | . | . | 1 | |  |
| 12 | . | . | . | . | . | . | . | . | . | . | C | G | C | . | . | . | . | . | . | . | . | . | . | . | . | . | . | . | . | . | . | . | . | . | . | . | . | . | . | . | . | 1 | |  |
| 13 | . | . | . | . | C | . | . | . | C | . | . | . | C | . | . | . | . | . | . | . | . | . | . | . | . | . | . | . | . | . | . | . | . | . | G | T | C | . | . | . | . | 1 | |  |
| 14 | . | . | . | . | C | . | . | . | C | . | . | . | C | . | . | . | . | . | . | . | C | . | . | C | . | . | . | . | . | . | . | . | . | . | G | T | C | . | . | . | . | 1 | |  |
| 15 | . | . | . | . | C | A | . | A | C | . | . | . | . | . | . | . | . | . | . | . | . | . | . | . | . | . | . | . | . | . | . | . | . | . | . | . | . | . | . | . | . | 1 | |  |
| 16 | . | . | . | . | C | A | . | A | C | . | . | . | C | . | . | . | . | . | . | . | . | . | . | . | . | . | . | . | . | . | . | . | . | . | . | . | . | . | . | . | . | 1 | |  |
| 17 | . | . | . | . | C | A | . | . | . | . | . | . | C | . | . | . | . | . | . | . | C | . | G | . | . | . | . | . | . | . | . | . | . | . | G | T | . | . | C | . | . | 1 | |  |
| 18 | . | . | . | . | C | A | . | . | . | . | . | G | C | . | . | . | . | . | . | . | . | . | . | . | . | . | . | . | . | . | . | . | . | . | . | . | . | . | . | . | . | 1 | |  |
| 19 | . | . | . | . | C | A | . | . | . | . | C | . | C | . | . | . | . | . | . | . | . | . | . | . | . | T | T | . | . | . | . | . | . | . | G | T | C | . | . | . | . | 1 | |  |
| 20 | . | . | A | . | C | A | . | A | . | . | . | . | C | . | . | . | . | . | . | . | . | . | . | . | . | . | . | . | . | C | . | . | . | . | . | T | C | A | . | . | T | 1 | |  |
| 21 | . | . | A | . | C | A | . | A | . | . | . | . | C | . | . | . | . | . | . | . | . | . | . | . | . | . | . | . | . | . | . | . | . | . | . | . | . | . | . | . | . | 1 | |  |
| 22 | . | . | A | . | C | A | . | A | . | . | C | . | C | . | . | . | . | . | A | T | C | . | . | . | . | . | . | . | . | . | . | . | . | . | G | T | C | . | . | . | . | 1 | |  |
| 23 | . | . | . | . | . | . | . | . | . | . | . | . | C | . | . | . | . | . | . | . | C | . | G | . | . | . | . | . | . | . | . | . | . | . | G | T | . | . | C | . | . | 1 | |  |
| 24 | . | . | . | . | . | . | . | . | . | . | . | . | C | . | . | . | . | . | . | T | C | . | G | . | T | T | T | . | . | . | . | . | . | . | G | T | C | . | . | . | . | 1 | |  |
| 25 | . | . | . | . | C | A | . | A | C | . | . | . | C | . | . | . | . | C | . | . | . | . | . | . | T | T | T | . | . | . | . | . | . | . | G | T | . | . | C | . | . | 1 | |  |
| 26 | . | . | . | . | C | A | . | A | C | . | . | . | C | T | . | . | . | . | . | . | C | . | . | . | . | . | . | . | . | . | . | . | . | . | G | T | . | . | C | . | . | 1 | |  |
| 27 | . | . | . | . | . | . | . | . | C | . | . | . | . | . | . | . | . | . | . | . | . | . | . | . | . | . | . | . | . | . | . | . | . | . | G | T | C | . | . | . | . | 1 | |  |
| 28 | C | . | . | . | . | . | . | . | . | . | . | . | . | . | . | . | . | C | . | . | . | . | . | . | T | T | T | . | . | . | . | . | . | A | G | T | C | . | . | T | . | 1 | |  |
| 29 | . | . | . | . | C | A | . | . | . | . | . | . | . | . | . | . | . | . | . | . | C | . | G | . | T | T | T | . | . | . | . | . | . | . | G | T | C | . | . | . | . | 1 | |  |
| 30 | . | . | . | . | C | A | . | . | . | . | C | . | C | . | . | . | . | . | A | T | C | . | . | . | . | . | . | . | . | . | . | . | . | . | G | T | C | . | . | . | . | 1 | |  |
| 31 | C | . | . | . | . | . | . | . | . | C | . | . | C | . | . | . | . | . | . | . | C | . | G | . | . | . | . | . | . | . | . | . | . | . | G | T | . | . | C | . | . | 1 | |  |
| 32 | C | . | . | . | . | . | . | . | . | C | . | . | C | T | . | . | . | . | . | T | C | A | . | . | . | . | . | . | . | . | . | . | . | . | G | T | . | . | . | . | . | 1 | |  |
| 33 | . | . | . | . | C | A | . | . | C | . | . | . | . | . | . | . | . | . | . | . | C | . | . | . | . | . | . | . | . | . | . | . | . | . | G | T | C | A | . | . | . | 1 | |  |
| 34 | . | . | . | . | C | A | . | . | C | . | C | G | . | . | . | . | . | . | . | . | C | . | G | . | T | T | T | . | . | . | . | . | . | . | G | T | C | . | . | . | . | 1 | |  |
| 35 | . | . | A | . | C | A | . | A | . | . | . | . | C | . | . | . | . | . | . | T | C | . | G | . | T | T | T | . | . | . | . | . | . | . | G | T | C | . | . | . | . | 1 | |  |
| 36 | . | . | A | . | C | A | . | A | . | . | C | G | C | . | . | . | . | . | . | . | . | . | . | . | . | . | . | . | . | . | . | . | . | . | . | . | . | . | . | . | . | 1 | |  |
| 37 | . | . | . | . | C | A | . | . | . | . | . | . | C | . | . | . | . | . | . | T | C | . | G | . | T | T | T | . | . | . | . | . | . | . | G | T | C | . | . | . | . | 1 | |  |
| 38 | . | . | . | T | C | A | . | . | C | . | . | . | C | . | . | . | . | C | . | . | . | . | . | . | T | T | T | . | . | . | . | . | . | A | G | T | C | . | . | T | . | 1 | |  |
| 39 | . | . | . | . | . | . | . | . | C | . | . | . | . | . | . | . | . | . | . | . | . | . | . | . | T | T | . | . | . | G | . | T | . | . | G | T | C | . | . | . | . | 52 | | OCCIP_T OCCIP_Z DOM_FR DOM_Eng DOM_AC DOM_NZ OCCF |
| 40 | . | . | . | . | . | . | . | . | . | . | . | . | . | . | . | . | . | . | . | T | C | . | . | . | T | T | . | . | . | C | . | T | . | . | G | T | C | . | . | . | . | 14 | | OCCIP_T DOM_Eng OCCF |
| 41 | C | G | . | . | . | . | . | . | . | . | . | . | . | . | . | . | . | . | . | . | . | . | . | . | T | T | . | . | . | C | . | T | . | . | G | T | C | . | . | . | . | 1 | | OCCIP |
| 42 | C | G | . | . | . | . | . | . | . | . | . | . | C | . | . | . | . | . | . | . | . | . | . | . | T | T | . | . | . | C | . | T | . | . | G | T | C | . | . | . | . | 1 | |  |
| 43 | . | . | . | . | . | . | . | . | . | . | . | . | C | . | . | . | . | . | . | T | C | . | . | . | . | . | . | . | . | C | . | . | . | . | . | . | . | . | . | . | . | 2 | |  |
| 44 | . | . | . | . | . | . | . | . | . | . | . | . | C | . | . | . | . | . | . | T | C | . | . | . | . | . | . | . | . | . | . | . | . | . | . | . | . | . | . | . | . | 1 | |  |
| 45 | . | . | . | . | . | . | . | . | C | . | . | . | . | . | . | . | . | . | . | T | C | . | . | . | T | T | . | . | . | C | . | T | . | . | G | T | C | . | . | . | . | 1 | |  |
| 46 | . | . | . | . | . | . | . | . | C | . | . | . | C | . | . | . | . | . | . | . | . | . | . | . | . | . | . | . | . | . | . | . | . | . | . | . | . | . | . | T | T | 1 | |  |
| 47 | . | . | . | . | C | . | . | . | C | . | . | . | C | . | . | . | . | . | . | . | . | . | . | . | T | T | T | . | . | . | . | . | . | . | G | T | C | . | . | T | . | 1 | |  |
| 48 | . | . | . | . | C | . | . | . | . | . | . | . | . | . | . | . | . | . | . | . | . | . | . | . | T | T | . | . | . | C | . | T | . | . | G | T | C | . | . | . | . | 1 | |  |
| 49 | . | . | . | . | . | . | C | . | C | . | . | . | . | . | . | . | . | . | . | . | . | . | . | . | . | T | . | . | . | G | . | T | . | . | G | T | C | . | . | . | . | 1 | |  |
| 50 | . | . | . | . | . | . | C | . | . | . | . | . | C | . | . | . | . | . | . | T | C | . | . | . | . | . | . | . | . | G | . | . | . | . | . | . | . | . | . | . | . | 1 | |  |
| 51 | . | . | . | . | . | . | . | . | C | . | . | . | . | . | . | C | . | . | . | . | . | . | . | . | T | T | . | . | . | G | . | T | . | . | G | T | C | . | . | . | . | 1 | |  |
| 52 | . | . | . | . | . | . | . | . | C | . | . | . | . | . | . | . | . | . | . | . | . | . | . | . | T | T | . | . | . | . | . | T | . | . | G | T | C | . | . | . | . | 1 | |  |
| 53 | . | . | . | . | . | . | . | . | C | . | . | . | . | . | . | . | . | . | . | . | . | . | . | . | T | T | T | . | . | . | . | . | . | . | G | T | C | . | . | T | . | 1 | |  |
| 54 | . | . | . | . | . | . | . | . | C | . | . | . | C | . | . | . | . | . | . | T | C | . | . | . | T | . | . | . | . | . | . | T | . | . | G | T | C | . | . | . | . | 2 | |  |
| 55 | . | . | . | . | . | . | . | . | . | . | . | . | . | . | . | . | . | . | . | . | . | . | . | . | T | T | . | . | . | . | . | T | . | . | G | T | C | . | . | . | . | 1 | |  |
| 56 | . | . | . | . | . | . | . | . | . | C | . | . | C | . | . | . | . | . | . | . | . | . | . | . | . | T | . | . | . | . | . | T | . | . | G | T | C | . | . | . | . | 1 | |  |
| 57 | . | . | . | . | C | A | . | . | . | C | . | . | C | . | . | . | . | . | . | T | C | . | . | . | . | . | . | . | . | . | . | . | . | . | . | T | . | . | . | . | . | 3 | |  |
| 58 | . | . | . | . | . | . | C | . | C | . | . | . | . | . | . | . | . | . | . | . | . | . | . | . | T | T | . | . | . | G | . | T | . | . | G | T | C | . | . | . | . | 1 | |  |
| 59 | . | . | . | . | . | . | . | . | C | . | . | . | . | . | . | . | . | . | . | . | . | . | . | . | T | T | T | . | G | . | . | . | . | . | G | T | C | . | . | T | . | 1 | |  |
| 60 | . | . | . | . | . | . | . | . | C | . | . | . | . | . | . | . | . | . | . | . | . | . | . | . | T | T | . | . | . | . | . | T | . | . | G | T | C | . | . | T | . | 1 | |  |
| 61 | . | . | . | . | . | . | C | . | C | . | . | . | . | . | . | . | . | . | . | . | . | . | . | . | T | T | T | . | . | . | . | . | . | . | G | T | C | . | . | T | . | 1 | |  |
| 62 | . | . | . | . | . | . | . | . | C | . | . | . | . | . | . | . | . | . | . | . | . | . | . | . | T | T | . | . | . | . | . | . | . | . | . | . | . | . | . | . | . | 1 | |  |
| 63 | . | . | . | . | . | . | . | . | C | . | . | . | . | . | . | . | . | . | . | . | . | . | . | . | . | T | . | . | . | C | . | T | . | . | G | T | C | . | . | . | . | 1 | |  |
| 64 | . | . | . | . | . | . | . | . | C | . | . | . | . | . | . | . | . | . | . | . | . | . | . | . | . | T | . | . | . | G | . | T | . | . | G | T | C | . | . | . | . | 1 | |  |
| 65 | . | . | . | . | . | . | . | . | C | . | . | . | . | . | . | . | . | . | . | . | . | . | . | . | . | T | . | . | . | . | . | T | . | . | G | T | C | . | . | . | . | 2 | |  |
| 66 | . | . | . | . | . | . | . | . | C | . | . | . | . | . | . | . | . | . | . | . | . | . | . | . | . | T | T | . | . | . | . | . | . | . | G | T | C | . | . | T | . | 1 | |  |
| 67 | . | . | . | . | . | . | . | . | C | . | . | . | . | . | . | . | . | . | . | . | C | . | . | . | T | T | . | . | . | G | . | T | . | . | G | T | C | . | . | . | . | 1 | | DOM_Eng |
| 68 | . | . | . | . | . | . | . | . | . | . | . | . | . | . | . | . | . | . | . | . | C | . | . | . | T | T | . | . | . | C | . | T | . | . | G | T | C | . | . | . | . | 1 | |  |
| 69 | . | . | . | . | . | . | . | . | . | . | . | . | C | . | G | . | . | . | . | T | C | . | . | . | . | . | . | . | . | . | . | . | . | . | . | . | . | . | . | . | . | 11 | | OCCF |
| 70 | . | . | . | . | . | . | . | . | C | . | . | . | C | . | . | . | C | . | . | . | . | . | . | . | T | T | T | . | . | G | T | T | . | . | G | T | C | . | . | . | . | 4 | |  |
| 71 | . | . | . | . | C | A | . | . | . | . | . | . | . | . | . | . | . | . | . | T | C | . | . | . | T | T | . | . | . | C | . | T | . | . | G | T | C | . | . | . | . | 1 | |  |
| 72 | C | . | . | . | . | . | . | . | . | . | . | . | C | . | G | . | . | . | . | T | C | . | . | . | . | . | . | . | . | . | . | . | . | . | . | T | . | . | . | . | . | 1 | |  |
| 73 | C | . | . | . | . | . | . | . | . | . | . | . | C | . | G | . | . | . | . | T | C | . | . | . | . | . | . | . | . | . | . | . | . | . | . | . | . | . | . | . | . | 2 | |  |
| 74 | . | . | . | . | . | . | . | . | C | . | . | . | C | . | . | . | C | . | . | . | . | . | . | . | T | T | T | . | . | G | . | T | . | . | G | T | C | . | . | . | . | 1 | |  |
| 75 | . | . | . | . | . | . | . | . | C | . | . | . | . | . | . | . | C | . | . | . | . | . | . | . | T | T | T | . | . | G | . | T | . | . | G | T | C | . | . | . | . | 1 | |  |

^*^OCA, *O. c. algirus*; OCC, *O. c. cuniculus*; IP, Iberian Peninsula; F, France ; T, Tarragona, Spain; Z, Zaragoza, Spain; DOM, Domestic breed; Eng, English Spot; AC, Argent Champagne; NZ, New Zealand; FR, French Lop
