# Supplementary material for: Insights into the European rabbit (Oryctolagus cuniculus) innate immune system: genetic diversity of the toll-like receptor 3 (TLR3) in wild populations and domestic breeds
Source: BMC Genet. 2013 Aug 21;14:73. doi: 10.1186/1471-2156-14-73 (PMC3844586; doi:10.1186/1471-2156-14-73)
Supplement: Additional file 3: Table S3 — Amplification conditions of TLR3. Characterisation of the conditions used for TLR3 amplification. Positions are according to the rabbit TLR3 sequence (ENSOCUG00000017763). [file 1471-2156-14-73-S3.docx]

**Table S3.** Characterisation of the conditions used for *TLR3* amplification. Positions are according to the rabbit *TLR3* sequence (ENSOCUG00000017763).

| **Exon** | **Identification** | **Primer sequence (5’ – 3’)** | **Position** | **Fragment length** |
| --- | --- | --- | --- | --- |
| 1 | OcTLR3_Fw1 | ATGAGCCAGAGTGTGCCTTATC | 1 | 510bp |
|  | OcTLR3_Rv1 | CTTGAAGATTTAACATTGGATC | 510 |  |
| 2 | OcTLR3_Fw2 | AGTGAAGTGACGATGTGCTG | 5949 | 453bp |
|  | OcTLR3_Rv2 | ACCAATACCCCAAGGCAGTA | 6402 |  |
| 3 | OcTLR3_Fw3 | GGCCTCTTTCTGAACAATGC | 9057 | 643bp |
|  | OcTLR3_Rv3 | CCAATGTCAAGCACCTCTAG | 9700 |  |
|  | OcTLR3_Fw4 | CCTTACTCATACTCAACCTG | 9607 | 601bp |
|  | OcTLR3_Rv4 | GTCATCAAAGACAGATTGTGG | 10208 |  |
|  | OcTLR3_Fw5 | CTTGGATGAGATCCCAGAAG | 10103 | 765bp |
|  | OcTLR3_Rv5 | TTTGCATAATGGGTCCTTTAG | 10868 |  |
| 4 | OcTLR3_Fw6 | ATTGAGGAAAATACATAACCTGA | 11300 | 466bp |
|  | OcTLR3_Rv6 | TTATGATAGAATCCCGTCACC | 11766 |  |
